# Quantile-based fecal hemoglobin concentration for assessing colorectal neoplasms with 1,263,717 Taiwanese screenees

**DOI:** 10.1186/s12911-019-0812-1

**Published:** 2019-05-02

**Authors:** Szu-Min Peng, Han-Mo Chiu, Hsiao-Hsuan Jen, Chen-Yang Hsu, Sam Li-Sheng Chen, Sherry Yueh-Hsia Chiu, Amy Ming-Fang Yen, Jean Ching-Yuan Fann, Yi-Chia Lee, Hsiu-Hsi Chen

**Affiliations:** 1 Institute of Epidemiology and Preventive Medicine, College of Public Health, National Taiwan University, Room 533, 5F. No. 17, Hsu Chow Road, Zhongzheng District, Taipei, 100 Taiwan; 2 Departments of Internal Medicine, National Taiwan University Hospital, No.7, Chung-Shan South Road, Zhongzheng District, Taipei, 100 Taiwan; 3 School of Oral Hygiene, College of Oral Medicine, Taipei Medical University, No. 250, Wu-Hsing Street, Sinyi District, Taipei, 110 Taiwan; 4 Department of Health Care Management and Healthy Aging Research Center, Chang Gung University, Taoyuan, Taiwan; 5 Division of Hepatogastroenterology, Department of Internal Medicine, Kaohsiung Chang Gung Memorial Hospital, Kaohsiung, Taiwan; 6 Department of Health Industry Management, School of Healthcare Management, Kainan University, No.1, Kainan Road, Luzhu District, Taoyuan, 338 Taiwan

**Keywords:** Colorectal cancer, Screening, Fecal hemoglobin

## Abstract

**Background:**

Although fecal hemoglobin concentration (f-Hb) was highly associated with the risk of colorectal neoplasms, current studies on this subject are hampered by skewedness of the data and the ordinal property of f-Hb has not been well studied yet. Our aim was to develop a quantile-based method to estimate adjusted percentiles (median) of fecal hemoglobin concentration and their derived prediction for the risk of multistage outcomes of colorectal disease.

**Methods:**

We used a 6-year follow-up cohort of Taiwanese nationwide colorectal screening program with fecal immunochemical testing (FIT) to obtain fecal hemoglobin concentration and applied accelerated failure time multi-variable analyses to make the comparison of adjusted median and other percentitles of fecal hemoglobin across four categories of colorectal carcinogenesis. We then predicted the risk of colorectal neoplasms on the basis of the corresponding percentile values by using accelerated failure time model with Bayesian inversion method.

**Results:**

The adjusted median fecal hemoglobin concentration of nonadvanced adenoma, advanced adenoma, and colorectal cancer were 57, 82, and 163 μg/g feces as opposed to 0 μg/g feces for the normal group. At 90 μg/g of f-Hb, the highly suspected cut-off for colorectal disease, the risks were 17% for non-advanced adenoma, 6% for advanced adenoma, and 9% for CRC. Life-time risks of each colorectal neoplasm were derived by percentiles of fecal hemoglobin concentration.

**Conclusion:**

Covariate-adjusted risk stratification for multistage outcomes of colorectal neoplasia were provided by using the quantiles of fecal hemoglobin concentration, yielding the estimated life-time risks of 25th to 75th quantitles, ranging from 0.5 to 44% for colorectal cancer, 0.2 to 46% for non-advanced adenoma, and 0.1 to 20% for advanced adenoma.

**Electronic supplementary material:**

The online version of this article (10.1186/s12911-019-0812-1) contains supplementary material, which is available to authorized users.

## Background

Quantitative fecal immunochemical testing (FIT) is widely used in population-based screening for early detection of colorectal neoplasms [1] and has the potential to reduce the mortality from colorectal cancer (CRC) [2–4]. The relationship between fecal hemoglobin concentration (f-Hb) and the risk for CRC, advanced-stage CRC, and mortality from CRC indicates that the increase of f-Hb with time (age) may parallel the growth of colorectal neoplasms [5–7]. While quantitative measurement of f-Hb has been considered to predict colorectal neoplasia [7–15], how to deal with skewness and ordinal feature of f-Hb has not been well elucidated. The log-transformed value of f-Hb for dealing with skewness may not follow normal distribution as seen in other similar biomarkers reported in previous studies [16, 17], It is therefore interesting to use an alternative quantile-based survival model to first estimate different percentiles of f-Hb concentration (f-Hb_p_) in feces across colorectal neoplasia groups by treating the ranking of f-Hb as that of survival time.

Moreover, predicting the risk for colorectal neoplasia using the conventional method that treats the disease status as the outcome and f-Hb as an independent variable with adjustment for relevant factors is not appropriate for the underlying factor (such as f-Hb) that is also a part of procedure related to the confirmation of disease as seen in our population-based screening for CRC with FIT that measures f-Hb.

Therefore, we proposed the Bayesian quantile-based survival method to first estimate covariate-adjusted f-Hb_50_ and f-Hb_p_ values and then to asses the life-time risk for the multistages of colorectal neoplasia by percentile-based f-Hb given the baseline risk of each colorectal neoplasm.

## Methods

### Study subjects

Our data were derived from the Taiwanese Nationwide Colorectal Cancer Screening Program, which used FIT as the screening tool. Details on the planning and implementation of the screening program have been described in full elsewhere [2, 3]. In brief, the nationwide screening program launched in 2004 provided a biennial FIT to all residents in Taiwan aged between 50 and 69 years. The target population consisted of a group of 5,417,699 subjects with a staggered entry into the program with the goal of a 20% coverage rate during the initial 5 years. During the study period, the program included 1,160,895 participants and achieved a coverage rate of 21.4% and a repeated screening rate of 28.3%. The f-Hb of each participant was measured using two brands of commercial kits (discussed below). Patients with positive results were referred for confirmatory diagnosis via colonoscopy as the major method. Individual information, including age, sex, family history of CRC, and brand of FIT test used, was obtained via questionnaire, and the outcomes regarding colorectal neoplasms were derived from the reports of the confirmatory diagnosis and cancer registry. The histopathology of colorectal neoplasms was classified according to the criteria of the World Health Organization [18].

Colorectal adenoma is categorized into non-advanced adenoma and advanced adenoma based on size and histological types, villous or dysplasia condition. If colorectal adenoma is larger than 10 mm in diameter or have a villous component or high-grade dysplasia, it is classified as advanced adenoma and otherwise non-advanced adenoma.

Participants with missing or unidentifiable FIT values or those for whom an unspecified method was used for the measurement of f-Hb were excluded from our analyses. Because we were interested in the f-Hb concentration just before disease diagnosis, we excluded CRC patients who had positive FIT results but were not compliant with orders for a colonoscopy or who did not participate in the repeated FIT screening after a negative colonoscopy.

### Bayesian quantile-based f-Hb for predicting the risk of colorectal neoplasia

To take into account the ordinal feature of f-Hb measured by each FIT test as mentioned earlier, we proposed the novel survival methodology with accelerated failure time (AFT) model on quantile-based f-Hb rather than interval-scaled f-Hb. To relive the concern over incident risk prediction excluding disease at baseline, we applied Bayesian inversion method to estimate incident baseline risk for colorectal neoplasia (prior) and to derive the posterior risk prediction for colorectal neoplasia by combining information on the percentiles of f-Hb by the disease status of colorectal neoplasia (likelihood). That means quantile-based f-Hb AFT model included colorectal neoplasia at baseline, but for predicting future risk with the Bayesian inversion method these cases at baseline were excluded.

In details, the proposed method therefore consists of two steps. First, using the concept of survival analysis, we ranked the value of f-Hb from the lowest to the highest to estimate the median and other percentiles of f-Hb corresponding to non-advanced adenoma, advanced adenoma, and invasive colorectal cancer by treating the value of f-Hb as survival time. The cumulative curve (the complementary survival curve) for the median and other quantiles of f-Hb were therefore plotted by reporting each 10th percentile values to reach different disease statuses of colorectal neoplasms. The adjusted median and other percentiles of f-Hb were estimated by using parametric survival model (see below) making allowance for age, gender, family history, and brands of FIT. In the second step, we then applied the Bayesian inversion method to derive posterior risk prediction for colorectal neoplasia based on two parts, baseline risk for colorectal neoplasm without using information on f-Hb but making allowance for other factors estimated by using Poisson regression model that is only based on incident cases by excluding colorectal neoplasia at baseline, and likelihood function using information with percentiles of f-Hb given the disease status of colorectal neoplasia derived from the first step.

### Data collection

#### Measurement of f-Hb

Patients’ f-Hb measurements were made using two brands of commercially available kits: the OC-Sensor (Eiken Chemical Co., Tokyo, Japan) and the HM-Jack (Kyowa Medex Co., Tokyo, Japan). With these 1-day methods, a single fecal sample was collected at home by each participant and then was sent to certified laboratories within 7 days. Quantitative FIT testing was performed at approximately 125 qualified laboratories nationwide. The cutoffs for the two kits were 100 ng/mL for the OC-Sensor and 8 ng/mL for the HM-Jack; the cutoff concentration in buffer for both tests could be transformed to a standardized reporting unit of 20 μg/g of feces [19]. The details have been previously reported [2].

#### Information obtained from questionnaires

Screening participants were asked to complete a questionnaire that was administered by face-to-face inquiry by the staff of the public health centers. The questionnaire solicited individual information about age, sex, and family history of CRC, which could be treated as confounding factors in the subsequent multivariable analysis.

### Confirmatory diagnosis

Patients who were screened and had positive FIT results were referred to receive a confirmatory diagnosis, mainly on the basis of a total colonoscopy or a sigmoidoscopy plus barium enema. Detailed confirmatory results, including size, location, and histopathology of colonic neoplasms, were recorded. Subjects who had negative FIT results were invited to participate in the next screening round.

### Statistical analysis

We first applied the conventional nonparametric method, the Kaplan-Meier method, to determine if there were differences between the presence or absence of colorectal neoplasms associated with the median value of f-Hb, after which we derived the cumulative distribution curve of percentile-based f-Hb by different disease statuses.

To adjust the covariates of interest, we applied the accelerated failure time (AFT) regression model by treating f-Hb as the time to event and disease status as the independent variable, with adjustments for age, sex, family history of CRC, and the brand of FIT used. We chose the Weibull distribution to fit our data. The most important reason we used the AFT model is that we want to estimate the f-Hb in every 10th percentile with the adjustment of covariates by different disease statuses. This information would be informative for clinical applications. It should be noted that as this is a periodical screening program with biennial FIT tests, f-Hb concentration used for analysis would be screen-round (time)-dependent. Namely, f-Hb at first screen may be different from that at second screen in the same individual. All repeated screening histories on f-Hb were all included in analysis. That means if the first screen is the negative FIT results its f-Hb value belongs to the normal group. If the second screen is detected as positive FIT and conformed as colorectal adenoma the f-Hb at second screen belongs to colorectal adenoma. The correlation of such multiple and repeated measurements on f-Hb and the corresponding disease status has been also accommodated in our AFT survival model.

**Fig. 1:**
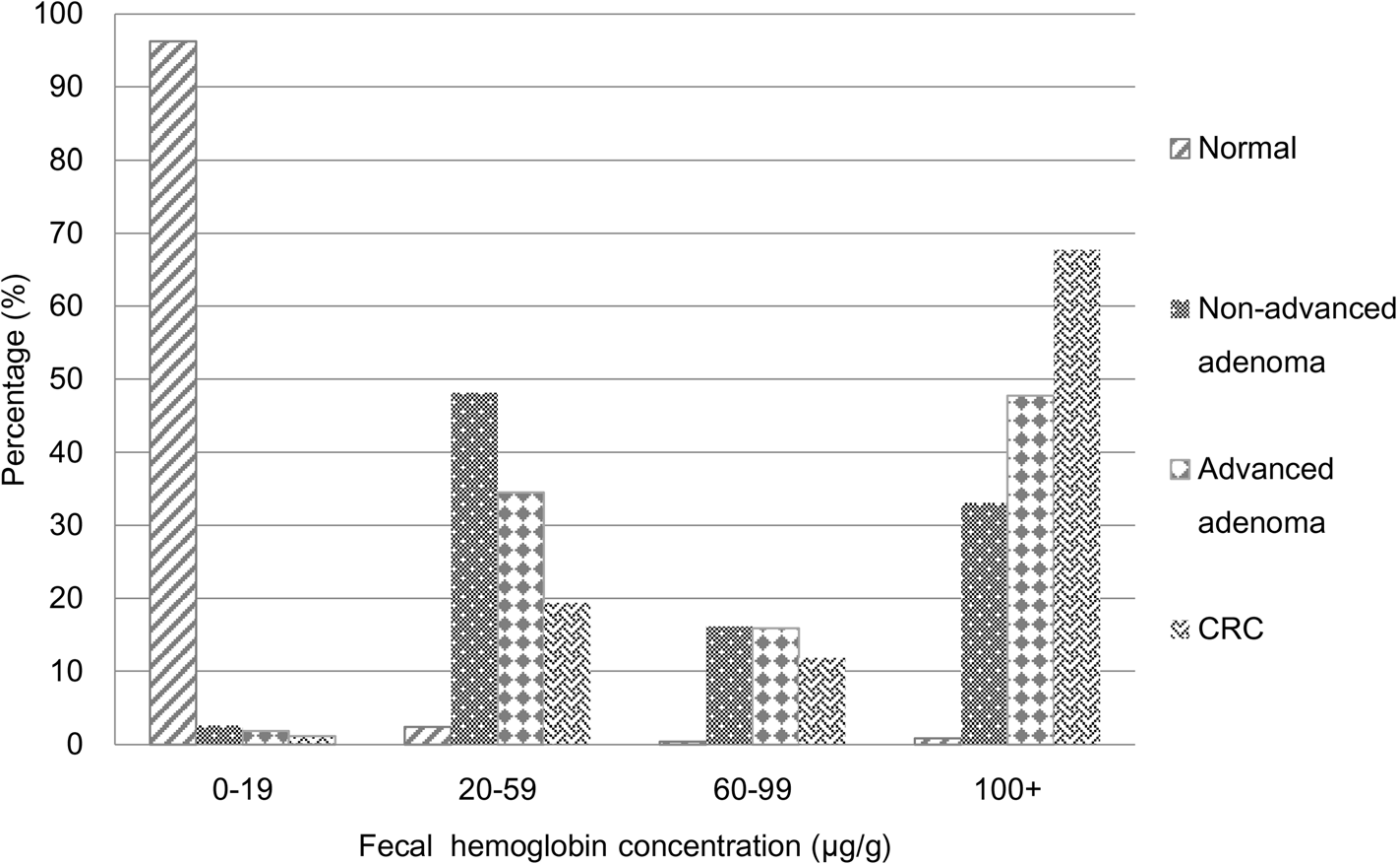
Ten hypothetical subjects illustrating the order of f-Hb (upper panel) and the corresponding distributions of f-Hb by the four groups of different disease statuses (lower panel)

Note that in terms of time ratio (TR) of being the value of f-Hb, an inverse relationship could be shown between the occurrence of colorectal neoplasms and f-Hb concentration. This means that although the rank of f-Hb was analogous to the rank of survival time, the TR, on average, would be the highest in the normal group, followed by the nonadvanced adenoma group, then the advanced adenoma, and the lowest in the CRC group. According to our intuitive hypothesis based on survival analyses, the higher the f-Hb, the lower the TR, but also the higher the risk for developing colorectal neoplasm (Fig. 1), we used the negative value of the coefficients estimated by the AFT model. Also, in order to solve the problem of undetected f-Hb, we added 0.5 units of each observation.

To predict life-time risk for colorectal neoplasia using the results of AFT model regarding the ranking of f-Hb as survival time as the likelihood, we applied Bayesian inversion method to combine this likelihood information on the percentile of f-Hb with prior information on the incidence of colorectal neoplasia to get posterior risk for colorectal neoplasia (the detailed elaboration is given in Additional file 1: Appendix).

Note that interval cancer patients (defined as invasive cancers diagnosed after a negative FIT and less than 2 years to the next screen) did not have information about f-Hb when diagnosed with cancer and turned out to be the censored data on f-Hb. To deal with the missing data, we calibrated the f-Hb of interval cancer cases from random samples of the prevalent and subsequent CRCs detected by screening matched with their corresponding sex and age at first screen using the cold-deck imputation method [20]. The reason of using imputation method for estimating the value of FIT for interval cancer is based on the two premises. The first is that the biological definition of interval cancer here is pursuant to the pathway of adenoma-carcinoma leading to the bleeding phenotype of interval cancer. Those interval cancers may be missed in the previous screen due to the undetectable bleeding phenotype stage and assume these undetectable bleeding phenotype interval cancers would grow up during inter-screening interval to become symptomatic bleeding phenotype as similar as the asymptomatic bleeding phenotype detected in the screen. The second is that as there are two components of interval cancer, the missed cases at prevalent screen (false negative cases) and the rapid progression of newly diagnosed cases after negative screen, the property of f-Hb for former may be therefore estimated from prevalent screen-detected cancers and that of f-Hb for the latter may be estimated from subsequent screen-detected cancers provided age (representing the maturation of tumour) and gender has been matched as we did here because both age and gender are two important factors in relation to time of onset and subsequent progression, and the sensitivity of FIT test.

## Results

### Descriptive data regarding the screening program

The final dataset for our analysis consisted of 1,028,859 prevalent screens and 1,263,717 repeated screens. The mean and median follow-up time of 1,028,859 subjects was 5.83 years and 5.67 years, respectively.

**Fig. 2:**
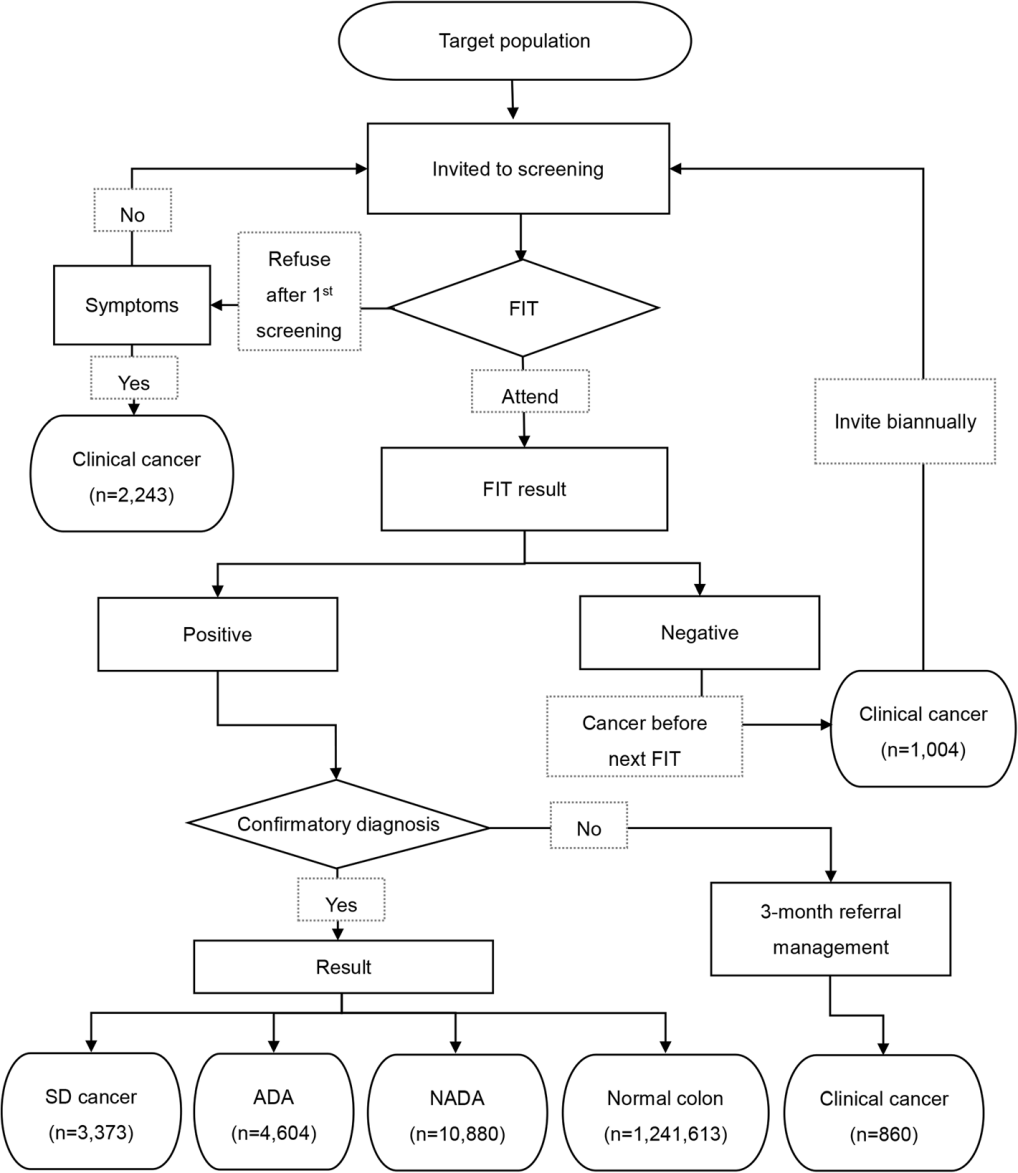
Flow chart of the nationwide colorectal cancer screening program (FIT = fecal immunochemical test; SD = screen-detected; ADA = advanced adenoma; NADA = non-advanced adenoma)

Figure 2 shows the process of CRC screening and the distribution of population screening data based on national periodical examinations of f-Hb levels, including the numbers of screening-detected CRC and clinically detected CRC, advanced adenoma, nonadvanced adenoma, and normal subjects. In screen-detected cancers, there were about 52.3% prevalent screen-detected cases and the rest were incident screen-detected cases. Although the longest repeated measures occurred five times, the subsequent screening rate remained low and resulted in fewer subsequent screen-detected cases (*n* = 1608).

Note that we elucidated the association between quantile f-Hb and colorectal neoplasia based on all data including 10,880 subjects (7814 identified at first screen and 3066 identified at subsequent screens) with non-advanced adenoma, 4604 subjects (3491 identified at first screen and 1113 identified at subsequent screens) with advanced adenoma, 1765 prevalent scree-detected CRC, 1608 subsequent screen-detected CRC, and 3247 interval CRC.

### Distribution of f-Hb

The f-Hb data of 1,263,717 screening participants stratified by their demographic characteristics are shown in Table 1, which shows a stepwise increase in the median and mean values of f-Hb as follows: normal colon, nonadvanced adenoma, advanced adenoma, and CRC. However, as expected, the f-Hb data were widely distributed. Male, older age, and positive family history of CRC were associated with higher f-Hb levels, and there were differences between the results from the different brands of FIT.

**Table 1:** Fecal hemoglobin concentration by disease statuses and demographic characteristics from the nationwide colorectal cancer screening program

Variable	Disease status	Number (%)	Median of f-Hb	Mean of f-Hb	SD of f-Hb	IQR of f-Hb
Status	Normal colon	1241613 (98.2)	0.0	8.1	400.4	2.4
	Non-advanced adenoma	10880 (0.9)	58.8	222.6	2381.2	109.8
	Advanced adenoma^a^	4604 (0.4)	92.0	254.6	616.1	205.7
	Colorectal cancer	6620 (0.5)	198.2	507.3	1366.5	327.2
Sex	Male	470810 (37.3)	0.2	18.1	652.4	3.0
	Female	792907 (62.7)	0	10.6	311.2	2.4
Age in years	50–54	395404 (31.3)	0	9.3	179.7	2.2
	55–59	359892 (28.5)	0	13.0	555.1	2.4
	60–64	250841 (19.8)	0.2	15.0	458.1	2.8
	65–69	257580 (20.4)	0.25	19.0	625.9	3.2
Family history of colorectal cancer	Yes	5638 (0.4)	0	22.3	212.1	2.4
	No	1258079 (99.6)	0.2	13.4	469.2	2.5
Brand of FIT	OC-Sensor	968870 (76.7)	0	7.6	115.4	1.6
	HM-Jack	294847 (23.3)	2	32.7	946.6	5.3
Overall		1263717	0.2	13.4	468.4	2.5

Abbreviation: *f-Hb* fecal hemoglobin concentration in μg/g, *SD* standard deviation, *IQR* interquartile range

^a^Defined as an adenoma of ≥10 mm in diameter or having a villous component or high-grade dysplasia

The distributions of the original f-Hb and the log-transformed data among the four groups are shown in Additional file 1: Figure S1. The log-transformed values improved positively skewed distribution of raw data but still did follow normal distribution.

### Univariable and multivariable regression analyses

Using the AFT model, the results of univariable and multi-variable analysis are listed in Additional file 1: Table S1. The results of the latter show the adjusted TR was higher in men than women (1.10, 95% CI: 1.09–1.10); the older age group had significantly higher f-Hb than the younger age group; subjects with a family history of CRC had higher f-Hb (1.08, 95% CI: 1.05–1.11); and a significant difference was noted between the results obtained from different brands of FIT (0.51, 95% CI: 0.51–0.52). Regarding disease status, after adjustment for other covariates (age, sex, family history, and brand of FIT), compared to the normal group, the adjusted TRs and 95% CIs of the non-advanced adenoma, advanced adenoma, and CRC groups were 9.13 (95% CI: 8.96–9.30), 11.35 (95% CI: 11.03–11.69), and 17.02 (95% CI: 16.61–17.44), respectively. These results clearly show that those who were diagnosed with CRC tended to have a significantly higher f-Hb level at screen, followed by the advanced adenoma and the non-advanced adenoma. Patients who were screened and had higher f-Hb levels also had a higher probability of being diagnosed with colorectal diseases.

### Medians and percentiles of f-Hb associated with colorectal neoplasms

**Fig. 3:**
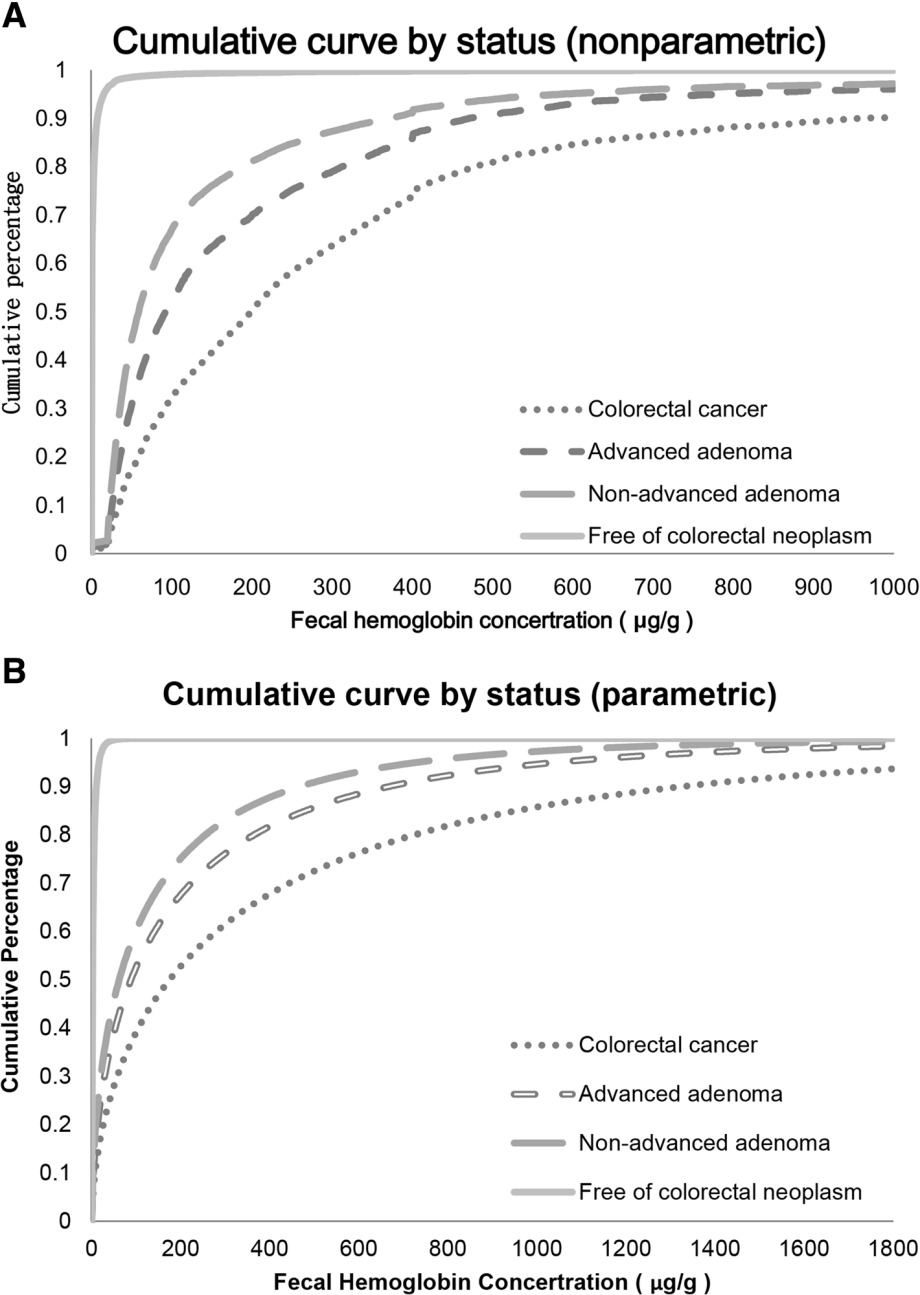
Cumulative percentage curves of f-Hb concentration among different disease statuses after correction: **a** the non-parametric Kaplan-Meyer method; **b** the parametric accelerated failure time model with a Weibull distribution

The cumulative curves of f-Hb levels by disease status are shown in Fig. 3 (a) (the nonparametric method: Kaplan–Meyer method) and Fig. 3 (b) (the parametric method: AFT model) without and with adjustment for covariates. In addition to the cumulative curves, the median and different percentiles per 10% increase in f-Hb levels (based on both nonparametric and parametric methods), are shown in Table 2.

**Table 2:** Median and percentiles of fecal hemoglobin concentration corresponding to colorectal non-advanced and advanced adenoma, and colorectal cancer based on the nonparametric and parametric methods

Percentile	Nonparametric method^a^			Parametric method^a^		
	Non-advanced adenoma	Advanced adenoma	Colorectal cancer	Non-advanced adenoma	Advanced adenoma	Colorectal cancer
10th	23.0^b^	25.8	33.8	2.4	3.4	6.8
20th	28.6	35.8	58.6	8.4	12.2	24.1
25th	32.0	41.6	72.8	12.9	18.7	37.0
30th	35.8	49.0	90.6	18.6	26.8	53.2
40th	45.3	66.8	140.0	34.0	49.2	97.5
50th	58.8	92.0	198.2	57.0	82.4	163.1
60th	78.8	129.0	267.7	91.2	131.9	261.2
70th	111.7	200.0	360.8	144.6	209.0	414.1
75th	141.8	247.3	400.0	183.5	265.2	525.3
80th	186.1	311.8	476.6	236.0	341.1	675.7
90th	368.4	468.6	951.4	431.9	624.2	1236.4

^a^Kaplan-Meyer method is used for the nonparametric method and the accelerated failure time model is used for the parametric method

^b^The unit of fecal hemoglobin concentration is μg/g

The median values (f-Hb_50_) were estimated as 59 μg/g, 92 μg/g, and 198 μg/g for non-advanced adenoma, advanced adenoma, and CRC, respectively, using the nonparametric method. When we used the parametric method (AFT model) to make adjustment for age, gender, brand of FIT kits, and family history, the corresponding results were 57 μg/g, 82 μg/g, and 163 μg/g, respectively. The estimates from the parametric method were lower than those from the nonparametric method within the 10th to 50th percentiles but became higher within the 60th to 90th percentiles. Additionally, other percentiles for different outcomes of colorectal neoplasms are also shown. For example, the 80th percentile values of f-Hb estimated by the AFT model were 236 μg/g, 341 μg/g, and 676 μg/g, respectively, for non-advanced adenoma, advanced adenoma, and CRC. Based on the results as shown in Table 2, the life-time risk prediction for colorectal neoplasia can be calculated by using Bayesian inversion method as shown in Table 3.

**Table 3:** Risk (in percentage, %) of colorectal neoplasia by median and percentiles of fecal hemoglobin concentration calculated by the accelerated failure time model

f-Hb (μg/g)	Non-advanced Adenoma	Advanced Adenoma	Colorectal Cancer
10th	< 0.1	< 0.1	< 0.1
20th	0.1	0.1	0.2
25th	0.2	0.1	0.5
30th	0.3	0.2	1.6
40th	0.9	0.9	11.9
50th	4.0	4.8	29.5
60th	17.3	15.1	35.8
70th	41.4	19.5	41.0
75th	45.5	19.7	44.3
80th	45.0	19.7	48.3
90th	39.0	19.0	60.7

It can be inferred that an increase in f-Hb percentile from 25th to 75th implies 88.6 times increase in the risk for CRC. On the other hand, the risk given each f-Hb_50_ obtained from Table 3 was 4.0% for non-advanced adenoma, 4.8% for advanced adenoma, and 29.5% for CRC. Besides, the risk of each disease status by the corresponding f-Hb level is also shown in Additional file 1: Table S5. It can be seen that at 90 μg/g of f-Hb, the highly suspected cut-off for colorectal neoplasia, the risks were 17% for non-advanced adenoma, 6% for advanced adenoma, and 9% for CRC.

## Discussion

The innovations of this study include both methodological and practical aspects. The development of a good methodology provides an unbiased evaluation of the association between the patient’s disease status during colorectal carcinogenesis and the ordinal outcome data of f-Hb. Based on these data, the drawback of using traditional statistical method is that the result is easily affected by the tail distributions of extreme values. To address this problem, we considered the f-Hb concentrations as the ordinal data and used survival analyses to assess the difference in the values of f-Hb across four disease groups with adjustments for covariates of interest. In this study, we ranked f-Hb concentration like the way of ranking time in an order. This justifies the use of survival model and its regression model to assess how the disease status affects the rank of f-Hb, which is exactly the same as how different risk groups affect the ranking of time to death as often seen in the survival analysis. Namely, ranking the order of f-Hb is analogous to ranking the order of time to event and time order is tantamount to f-Hb order. Since short time to event leads to a higher hazard but low f-Hb leads to a lower hazard ratio. Therefore, the time (f-Hb) ratio here indicates the negative hazards ratio. The higher the time ratio is the higher the f-Hb is.

The application of this new methodology to f-Hb values obtained from FIT-based population-based screening not only renders the application of FIT-based data become available for international comparison but also offers useful information that may aid health decision-makers designing a customized screening policy for personalized preventive strategies based on the percentile or true value of f-Hb.

### The comparison of the findings with those from published studies

Evaluation of the ordinal property of a biomarker such as f-Hb that is measured by FIT and is widely used for population-based CRC screening is difficult because, in addition to the skewed property of such an ordinal data, the dynamic nature of f-Hb during the repeated FIT test, the multistage outcome of colorectal neoplasm, and the relationships of the upper and lower limits to the status of colorectal neoplasm render the elucidation of the dynamics of f-Hb very intractable. To consider these intractable issues, we used a simplified statistical approach with the AFT model that regarded the f-Hb as the dependent variable of time to event. The disease statuses, including normal colon, non-advanced adenoma, advanced adenoma, and CRC, were the main independent variables of interest, making allowance for age, sex, family history of CRC, and brand of FIT used. We also took them into account when we applied Bayesian inversion method to predict the corresponding risk of colorectal neoplasm.

Although f-Hb concentration has been already shown to predict the occurrence incident colorectal neoplasm and mortality of colorectal cancer, treating the value of f-Hb as ordinal data type has been scarcely addressed until several recent studies have found the feasibility of treating f-Hb as ordinal data type rather than only the dichotomous variable for the use in a qualitative manner. Garcia et al. found that f-Hb value was significantly higher in the severest group of colorectal lesion [21]. Kim et al., limited to FIT positive case (f-Hb≧20 μg/g), analyzed f-Hb as quartiles and found that higher quartile was significantly associated with advanced stage colorectal neoplasm [22]. These findings support the use of f-Hb in the manner of ordinal data.

Instead of regarding the f-Hb concentrations as the covariates and the colorectal diseases as dependent variables, we applied the survival regression model to relate the disease status to the variations in f-Hb levels. Modeling the concentrations of f-Hb in this manner may not only elucidate the disease progression of colorectal neoplasms but also may provide a new insight into how the percentiles (median) of f-Hb are associated with the progression of colorectal adenoma and CRC. The estimated adjusted f-Hb_50_ by the AFT model was 163 μg/g for CRC, 82 μg/g for advanced adenoma, and 57 μg/g for nonadvanced adenoma. Our findings showed results similar to those of previous studies in the comparison of the median and mean f-Hb values between different disease groups. However, we also found the absolute median and mean values were heterogeneous across studies (Additional file 1: Table S2-S4). Caution should be taken to interpret this heterogeneous finding as the target population may be different from studies to studies. As seen in the Additional file 1: Table S4, the f-Hb_50_ in the previous Taiwanese Liao’s study [13] was much higher than ours (198.2 μg/g) because their target population was high risk group enrolled from hospital. Note that both Levi’s and Digby’s study [9, 12] showed a little higher in f-Hb concentration than ours whereas the rest of studies [8, 11, 15] showed lower f-Hb_50_. These findings indicate the heterogeneity of the f-Hb distribution when we analyzed a large repository of population-based screening data. While using the rank-based f-Hb concentration; that is percentiles, the corresponding life-time risks of certain disease group can be estimated and the percentiles become comparable between different areas and races.

### The limitations of the study

The first concern over this study is pertaining to the values of f-Hb before the diagnosis of interval cancers. Because it is not possible to know the exact value of f-Hb for interval cancer when some cases were missed at screening but surfaced during clinical treatment, the direct use of f-Hb measured during previous screens for interval cancers is not correct. The cold-deck method was used for filling in the missing values of f-Hb for these interval cancers. Figure 3 shows the corrected curve for CRC. This again underscores the complexity of the statistical properties of f-Hb measurements. However, this model is not yet good enough to provide a full picture of the dynamic changes of f-Hb measurements to identify interval cancers because we assumed the f-Hb levels of interval cancers that were missed at previous screenings may be captured by the next screen among screen-detected cases matched for age and sex given the premise of bleeding phenotype as indicated in the method section. However, interval cancers may also contain another non-bleeding phenotype as there are some colorectal cancer following de novo pathway with flat and possible non-bleeding pathway. They may not lead to the elevated f-Hb. As they are also not detected by screen and the percentage of de novo pathway is rare our results may not be substantially affected.

The second limitation is that as previous studies have shown that sensitivity of FIT for non-advanced adenomas is low the median f-Hb concentration for non-advanced adenomas is far higher than that for the normal group may be due to detection bias. That means that non-advanced adenomas with f-Hb concentrations below 20 would be misclassified as the normal subjects. The quantile-based method making allowance for such a misclassification is the subject of ongoing research.

### Implications of screening and surveillance policy for colorectal cancer

From the practical aspect of screening, the empirical findings presented here provide new insights into policy-making for CRC screening and surveillance of early CRC detected with FIT. These results also facilitate a better understanding of the f-Hb_50_ value and the threshold of f-Hb for different outcomes and can be used as a priority-setting indicator for colonoscopy. Negative FIT could be further categorized into low risk or average-risk group based on f-Hb level. For those with lower f-Hb and lower risk might be able to prolong their inter-screening interval, which, in turn, reduces the clinical demand for colonoscopy. Even those with higher f-Hb with negative confirmatory diagnosis may be referred to take colonoscopy directly in the next round of screen. Finally, the risk stratification of f-Hb for non-compliance with colonoscopy is still very useful for the priority of colonoscopy as the previous study also demonstrates the dose-response relationship between f-Hb and mortality and advanced CRC for non-compliant.

## Conclusions

By treating f-Hb value as ordinal data, a quantile-based survival method has been performed to estimate covariate-adjusted median value and other percentiles by three levels of colorectal neoplasm, non-advanced adenoma, advanced adenoma, and colorectal cancer. Bayesian inversion method was further used to give posterior life-time risks for colorectal neoplasia by baseline risk of colorectal neoplasia in combination with information on the percentile of f-Hb by the disease status of colorectal neoplasia.

## Additional file

Additional file 1: Supplementary materials containing the detailed methodology of Bayesian inverse method, one appendix figure, and five appendix tables were included in the supplementary file. (DOCX 358 kb)

## Abbreviations

AFT: Accelerated failure time
CRC: Colorectal cancer
f-Hb: Fecal hemoglobin concentration
FIT: Fecal immunochemical testing
TR: Time ratio
