# Supplementary material for: Quantile-based fecal hemoglobin concentration for assessing colorectal neoplasms with 1,263,717 Taiwanese screenees
Source: BMC Med Inform Decis Mak. 2019 May 2;19:94. doi: 10.1186/s12911-019-0812-1 (PMC6498550; doi:10.1186/s12911-019-0812-1)
Supplement: Supplementary file 1 — Supplementary materials containing the detailed methodology of Bayesian inverse method, one appendix figure, and five appendix tables were included in the supplementary file. (DOCX 358 kb) [file 12911_2019_812_MOESM1_ESM.docx]

# Additional file 1

This file has been provided by the authors to give readers additional information about their work.

Supplement to: Peng SM, Chiu HM, Jen HH, et al. Rank-based Fecal Hemoglobin Concentration for Assessing Colorectal Neoplasms with 1,263,717 Taiwanese Screenees

| **Item** | **Description** |
| --- | --- |
| Bayesian inverse method | Detail methodology that support calculation of lifetime risk of colorectal neoplasm |
| Appendix Figure 1 | Histogram of f-Hb by four disease statuses: (1A) original f-Hb; (1B) logarithm transformed f-Hb. |
| Appendix Table 1 | Time ratios from the univariate and multivariate regression analyses for the fecal hemoglobin concentration |
| Appendix Table 2 | Comparisons of fecal hemoglobin concentration (μg/g) of nonadvanced adenoma |
| Appendix Table 3 | Comparisons of fecal hemoglobin concentration (μg/g) of advanced adenoma |
| Appendix Table 4 | Comparisons of fecal hemoglobin concentration (μg/g) of colorectal cancer |
| Appendix Table 5 | Conditional risk (in percentage, %) of fecal hemoglobin concentration (μg/g) of four statuses |

**Bayesian inverse method**

First, we applied an accelerated failure time (AFT) model to our data which included information on prevalent and repeated rounds of screen to estimate the percentile of f-Hb by type of colorectal neoplasia (denoted as *Dstatus*) to form likelihood function used below. We then employed a Bayesian inverse method given average values of adjusted covariates (X) used in the likelihood function and the prior of incidence of these diseases excluding disease at baseline (cases detected at prevalent screen) to estimate the risk of CRC and adenoma.

$$P\left( Dstatus|fHb,\boldsymbol{X} \right)=\frac{P\left( fHb|Dstatus,\boldsymbol{X} \right)\cdot P\left( Dstatus|\boldsymbol{X} \right)}{P\left( fHb,\boldsymbol{X} \right)}$$

The estimated disease incidence (estimated by a Poisson regression model with empirical data excluding disease at baseline) would be:

$P\left( CRC|\boldsymbol{X} \right)=$96.40/100,000 ; $P\left( Adv-adenoma|\boldsymbol{X} \right)=$53.66/100,000 ; $P\left( Nonadv-adenoma|\boldsymbol{X} \right)=$135.34/100,000 ;

where *Dstatus* represents disease types including CRC, advanced adenoma, and non-advanced adenoma. ***X*** were the covariates we adjusted in AFT model, including sex, age, family history of CRC, and brand of FIT.

## Appendix Figure S1. Histogram of f-Hb by four disease statuses: (1A) original f-Hb; (1B) logarithm transformed f-Hb.

| (1A) | 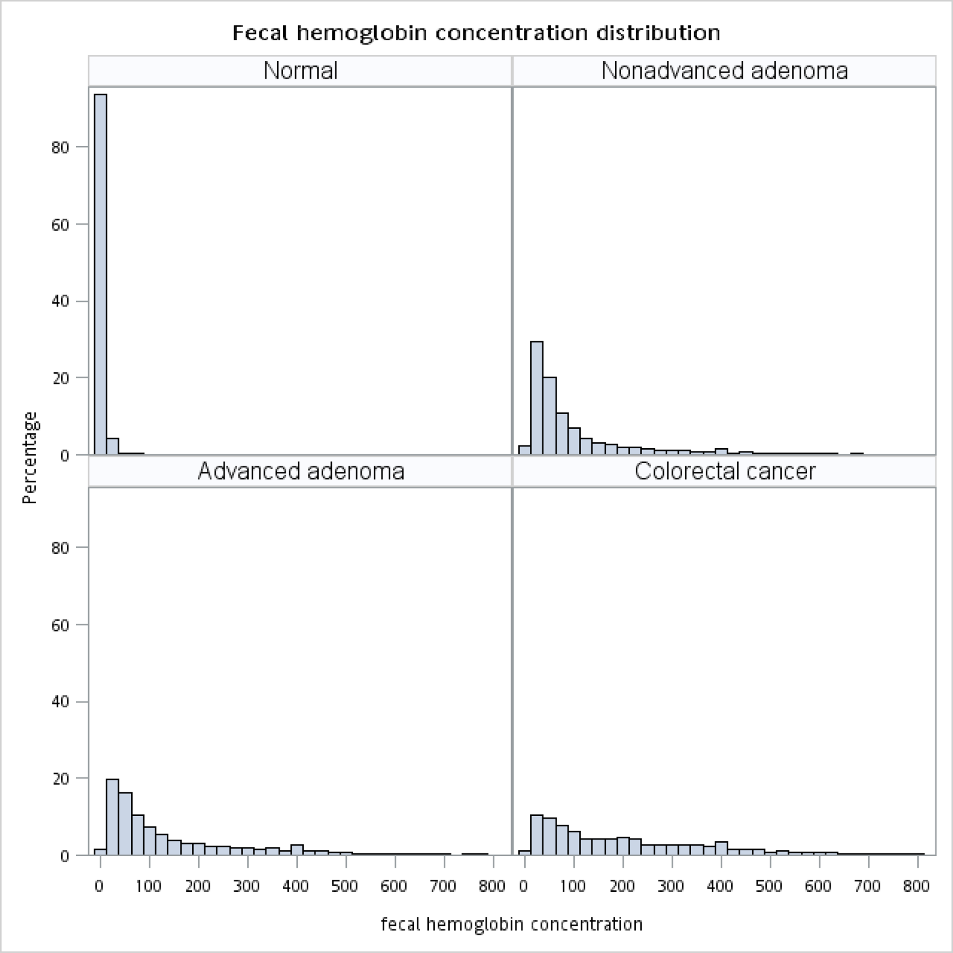 |
| --- | --- |
| (1B) | 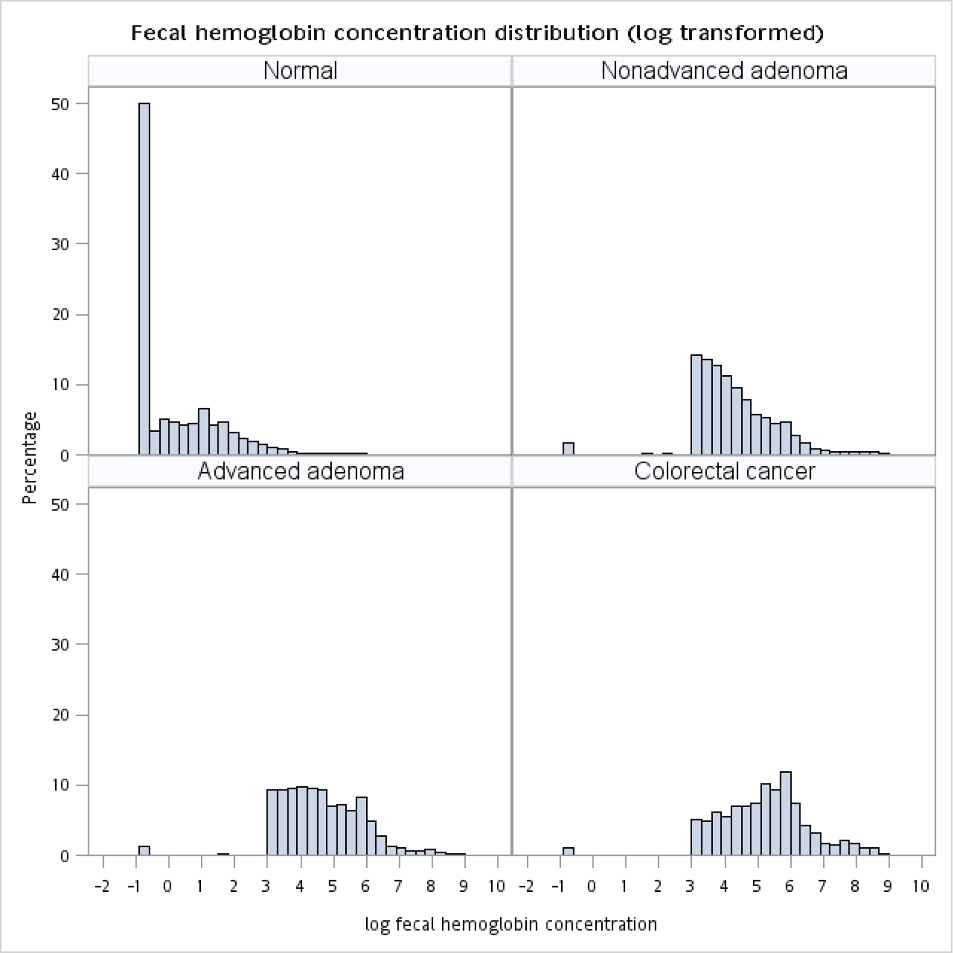 |

## Appendix Table S1. Time ratios from the univariate and multivariate regression analyses for the fecal hemoglobin concentration

|  |  | **Univariable** | **Multivariable*** |
| --- | --- | --- | --- |
| **Variable** |  | **Time ratio (95% CI)** | **Adjusted time ratio (95% CI)** |
| Disease status | Normal (reference) | 1 | 1 |
|  | Nonadvanced adenoma | 8.80 (8.63-8.96) | 9.13 (8.96-9.30) |
|  | Advanced adenoma | 10.61 (10.31-10.92) | 11.35 (11.03-11.69) |
|  | Colorectal cancer | 15.33 (14.96-15.70) | 17.02 (16.61-17.44) |
| Sex | Female (reference) | 1 | 1 |
|  | Male | 1.17 (1.17-1.18) | 1.10 (1.09-1.10) |
| Age in years | 50-54 (reference) | 1 | 1 |
|  | 55-59 | 1.07 (1.06-1.07) | 1.02 (1.02-1.03) |
|  | 60-64 | 1.17 (1.16-1.18) | 1.10 (1.09-1.10) |
|  | 65-69 | 1.26 (1.25-1.27) | 1.15 (1.15-1.16) |
| Family history of colorectal cancer | No (reference) | 1 | 1 |
|  | Yes | 1.18 (1.15-1.21) | 1.08 (1.05-1.11) |
| Brand of FIT^†^ | Brand 2 (reference) | 1 | 1 |
|  | Brand 1 | 0.57 (0.57-0.57) | 0.51 (0.51-0.52) |

* Multivariable accelerated failure time model with a Weibull shape parameter is 0.593.

^†^ Brand 1 = OC-Sensor; Brand 2 = HM-Jack.

## Appendix Table S2. Comparisons of fecal hemoglobin concentration (μg/g) of nonadvanced adenoma

| **Authors, year** | **Country** | **Brands of FIT** | **Case no.** | **Cut-off value** | **Median of f-Hb** | **Mean of f-Hb** | **SD of f-Hb** | **IQR of f-Hb** |
| --- | --- | --- | --- | --- | --- | --- | --- | --- |
| Levi et al. 2007 (8) | Israel | OC-Micro | 170 | 20 | 2.2 | 15.8 | 47.2 | 6.2 |
| Hol et al. 2009 (9) | Netherlands | OC-Sensor | 45 | 10 | 22.4 | – | – | – |
| Digby et al. 2013 (10) | UK | OC-Sensor | 119 | 80 | 152 | – | 13.7 | – |
| Liao et al. 2013 (11) | Taiwan | HM-Jack | 221 | 30 | 89.8 | 441.3 | 1259.6 | 210.3 |
| Ou et al. 2013 (12) | Taiwan | OC-Sensor | 132 | 20 | – | 4.5 | – | – |
| Auge et al. 2014 (13) | Spain | OC-Sensor | 548 | 20 | 42 | – | – | 66 |
| Hernandez et al. 2014 (14) | Spain | OC-Sensor | 202 | 15 | – | 8.5 | 44.9 | – |
| van Doorn et al. 2015 (15) | Netherlands | OC-Sensor | 195 | 10 | 19 | 50 | 95 | 23 |
| The present study | Taiwan | OC-Sensor or HM-Jack | 10,880 | 20 | 58.8 | 222.6 | 2,381.2 | 109.8 |

## Appendix Table S3. Comparisons of fecal hemoglobin concentration (μg/g) of advanced adenoma

| **Authors, year** | **Country** | **Brands of FIT** | **Case no.** | **Cut-off value** | **Median of f-Hb** | **Mean of f-Hb** | **SD of f-Hb** | **IQR of f-Hb** |
| --- | --- | --- | --- | --- | --- | --- | --- | --- |
| Levi et al. 2007 (8) | Israel | OC-Micro | 74 | 20 | 22.6 | 97 | 148.8 | 110.2 |
| Hol et al. 2009 (9) | Netherlands | OC-Sensor | 79 | 10 | 74.6 | – | – | – |
| Digby et al. 2013 (10) | UK | OC-Sensor | 190 | 80 | >200 | – | – | – |
| Liao et al. 2013 (11) | Taiwan | HM-Jack | 201 | 30 | 127.5 | 718.3 | 1751.1 | 385.3 |
| Ou et al. 2013 (12) | Taiwan | OC-Sensor | 39 | 20 | – | 37.6 | – | – |
| Auge et al. 2014 (13) | Spain | OC-Sensor | 1,147 | 20 | 92 | – | – | 242 |
| Hernandez et al. 2014 (14) | Spain | OC-Sensor | 92 | 15 | – | 46.6 | 108.6 | – |
| van Doorn et al. 2015 (15) | Netherlands | OC-Sensor | 332 | 10 | 34 | 87 | 127 | 76 |
| The present study | Taiwan | OC-Sensor or HM-Jack | 4,604 | 20 | 92.0 | 254.6 | 616.1 | 205.7 |

*Includes any advanced adenoma (i.e., lesions equal to10 mm or more in size or with a villous component or high-grade dysplasia) or 3 or more non-advanced adenomas.

## Appendix Table S4. Comparisons of fecal hemoglobin concentration (μg/g) of colorectal cancer

| **Authors, year** | **Country** | **Brands of FIT** | **Case no.** | **Cut-off value** | **Median of f-Hb** | **Mean of f-Hb** | **SD of f-Hb** | **IQR of f-Hb** |
| --- | --- | --- | --- | --- | --- | --- | --- | --- |
| Levi et al. 2007 (8) | Israel | OC-Micro | 17 | 20 | 209.4 | 217.4 | 164.2 | 231.4 |
| Hol et al. 2009 (9) | Netherlands | OC-Sensor | 16 | 10 | 80.8 | – | – | – |
| Digby et al. 2013 (10) | UK | OC-Sensor | 39 | 80 | >200 | – | – | – |
| Liao et al. 2013 (11) | Taiwan | HM-Jack | 48 | 30 | 747.3 | 2278.6 | 3131.8 | 3528.5 |
| Ou et al. 2013 (12) | Taiwan | OC-Sensor | 3 | 20 | – | 271.4 | – | – |
| Auge et al. 2014 (13) | Spain | OC-Sensor | 294 | 20 | 160 | – | – | 378 |
| Hernandez et al. 2014 (14) | Spain | OC-Sensor | 5 | 15 | – | 199.6 | 215.1 | – |
| van Doorn et al. 2015 (15) | Netherlands | OC-Sensor | 39 | 10 | 136 | 199 | 185 | 250 |
| The present study | Taiwan | OC-Sensor or HM-Jack | 6,620 | 20 | 198.2 | 507.3 | 1,366.5 | 327.2 |

## Appendix Table S5. Conditional risk (in percentage, %) of fecal hemoglobin concentration (μg/g) of three colorectal neoplasm related disease statuses

| **f-Hb**  **(μg/g)** | **Nonadvanced adenoma** | **Advanced adenoma** | **Colorectal cancer** |
| --- | --- | --- | --- |
| 1 | 0.03 | 0.01 | 0.01 |
| 10 | 0.11 | 0.04 | 0.05 |
| 20 | 0.31 | 0.11 | 0.14 |
| 30 | 0.71 | 0.25 | 0.32 |
| 40 | 1.43 | 0.51 | 0.68 |
| 50 | 2.66 | 0.96 | 1.31 |
| 60 | 4.64 | 1.70 | 2.36 |
| 70 | 7.58 | 2.81 | 3.99 |
| 80 | 11.62 | 4.37 | 6.30 |
| 90 | 16.65 | 6.34 | 9.29 |
| 100 | 22.30 | 8.58 | 12.78 |
| 120 | 33.04 | 13.00 | 19.93 |
| 150 | 42.53 | 17.24 | 27.53 |
| 180 | 45.39 | 18.91 | 31.36 |
| 200 | 45.65 | 19.36 | 32.86 |
| 300 | 43.01 | 19.71 | 37.21 |
| 400 | 39.91 | 19.57 | 40.52 |
| 500 | 37.12 | 19.35 | 43.54 |
| 600 | 34.59 | 19.07 | 46.34 |
| 700 | 32.28 | 18.76 | 48.96 |
| 800 | 30.16 | 18.41 | 51.43 |
